# Comprehensive and Quantitative Proteomic Analysis of Metamorphosis-Related Proteins in the Veined Rapa Whelk, *Rapana venosa*

**DOI:** 10.3390/ijms17060924

**Published:** 2016-06-15

**Authors:** Hao Song, Hai-Yan Wang, Tao Zhang

**Affiliations:** 1 Institute of Oceanology, Chinese Academy of Sciences, Qingdao 266071, China; songhao13@mails.ucas.ac.cn; 2 University of Chinese Academy of Sciences, Beijing 100049, China

**Keywords:** transcriptome, *Rapana venosa*, gastropod, larva, digital gene expression

## Abstract

Larval metamorphosis of the veined rapa whelk (*Rapana venosa*) is a pelagic to benthic transition that involves considerable structural and physiological changes. Because metamorphosis plays a pivotal role in *R. venosa* commercial breeding and natural populations, the endogenous proteins that drive this transition attract considerable interest. This study is the first to perform a comprehensive and quantitative proteomic analysis related to metamorphosis in a marine gastropod. We analyzed the proteomes of competent *R. venosa* larvae and post-larvae, resulting in the identification of 5312 proteins, including 470 that were downregulated and 668 that were upregulated after metamorphosis. The differentially expressed proteins reflected multiple processes involved in metamorphosis, including cytoskeleton and cell adhesion, ingestion and digestion, stress response and immunity, as well as specific tissue development. Our data improve understanding of the physiological traits controlling *R. venosa* metamorphosis and provide a solid basis for further study.

## 1. Introduction

The veined rapa whelk (*Rapana venosa*) is an economically important sea snail in China, and since 1992, there has been interest in its commercial aquaculture [1]. However, sea-ranching efforts have been hampered by difficulties cultivating larvae during the settlement and metamorphosis stages. In countries that do not consume *R. venosa*, such as the United States, Argentina, and France, this predatory species has become an invasive pest due to unintended worldwide transport and severely disrupts the survival of native bivalves [2,3,4,5,6]. Because *R. venosa* population dynamics and spatial expansion are dominated by recruitment and survival rate during metamorphosis, which is a vital process in the species’ biphasic life cycle, understanding the mechanisms behind this process is necessary for both successful aquaculture and invasion control. Moreover, the metamorphosis of *R. venosa* is unusual compared with other lifelong phytophagous gastropods for exhibiting considerable developmental specificity; the planktonic, pelagic larvae go from filter-feeding on microalgae to carnivorous juveniles that prey on bivalves [7]. This transition occurs rapidly, despite fundamental changes in morphology including velum degeneration and reabsorption, foot reorientation and elongation, as well as secondary-shell growth [7]. Thus, clarifying *R. venosa* metamorphosis is also of theoretical interest to gastropod researchers.

However, information about *R. venosa* metamorphosis is relatively scarce. A previous study had documented the morphological changes that occur during this process [7]. Additionally, CaCl_2_ and acetylcholine chloride were found to be effective and low-toxicity inducers of metamorphosis in *R. venosa* pelagic larvae [8], suggesting these compounds might be suitable for applying to its artificial seeding. Finally, a comprehensive transcriptomic profile has been constructed from *R. venosa* planktonic larvae and post-larvae [9], which paves the way for studies on metamorphosis-related gene activity. However, because complex gene regulation occurs during post-transcription and post-translation [10,11], proteomic data are required to provide more concrete support for conclusions based on transcriptome data. Indeed, proteomic analysis has been successfully applied to identify a number of metamorphosis-related proteins in marine-invertebrates, specifically in bryozoans [12], polychaetes [13,14], and barnacles [12,15]. To our knowledge, no proteomic study has been conducted to investigate gastropod metamorphosis.

Although two-dimensional electrophoresis (2DE) is the most common proteomic approach, the method lacks the sensitivity to identify low-abundance proteins or those not amenable to gels [16]. Moreover, 2DE’s accuracy is potentially compromised by the phenomenon of protein co-migration [17]. The recently developed, high-throughput isobaric tag for relative and absolute quantitation (iTRAQ) has therefore become increasingly popular. This method labels peptides with isobaric (same-mass) reagents consisting of reporter ions and their equalizing balance groups. During mass spectrometry (e.g., collision-induced dissociation (CID)), the reporter ions are then separated from the labelled peptides, allowing for determination of ion intensity and thus peptide quantity. Therefore, iTRAQ differs from other quantitative proteomics technologies, which tend to measure precursor (pre-fragmentation) ion intensities. The difference allows for greater accuracy and reliability [18]. In this current study, we chose iTRAQ to assess proteomic changes during metamorphosis via a comparative proteomic analysis on competent larvae and juveniles of *R. venosa*. We were able to identify and annotate over 5000 proteins through searching the *R. venosa* transcriptome with protein sequences [9]; 1138 of the identified proteins were differentially expressed, during metamorphosis, suggesting that they are responsible for the process. Our results showed that these differentially expressed proteins function in diverse biological processes, including cytoskeleton and cell adhesion, ingestion and digestion, stress response and immunity, as well as specific tissue development. These findings provide a proteomic overview of gastropod metamorphosis and facilitate future research on protein function during the transitions of a biphasic life cycle.

## 2. Results

### 2.1. General Characterization of Proteomic Data

**Figure 1:**
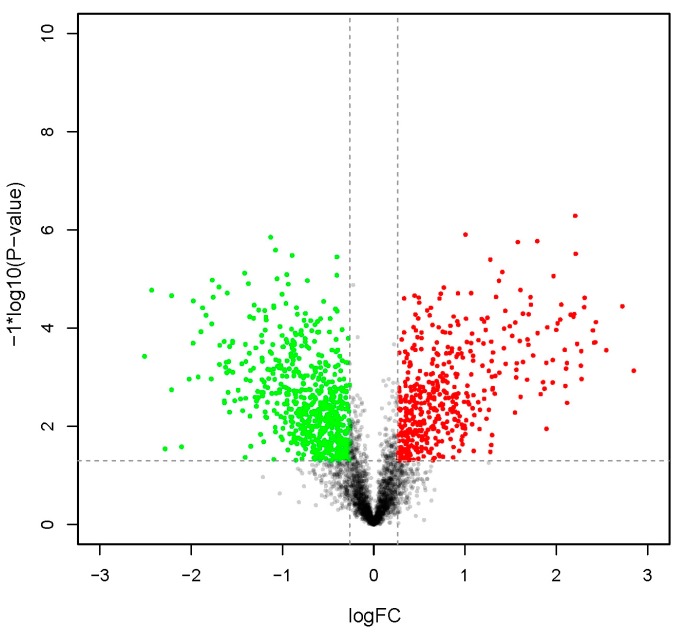
Change in global protein abundance between the post-larval stage (PL) and the competent larval stage (CL). LogFC represents log_2_Ratio (PL/CL); proteins with log_2_Ratio (PL/CL) >0.26 or <−0.26 are colored (**red** for fold changes >1.20 and **green** for <0.83).

Raw data have been deposited to the ProteomeXchange Database (accession number: PXD004119). As shown in Table 1, of the 224,473 detected spectra, 46,485 were considered unique. Moreover, 5321 proteins were identified. Figure 1 displays the overall changes to protein abundance before and after metamorphosis. More detailed information on these 5321 proteins is available in Table S1, while variation in expression during metamorphosis is shown in Table S2: 470 proteins were upregulated and 668 were downregulated after metamorphic transition (Table S2). Homologous sequence analysis of these differentially expressed proteins (DEPs) revealed four functional groups of interest (Table 2): cytoskeleton and cell adhesion, ingestion and digestion, stress response and immunity, as well as specific tissue development.

### 2.2. Functional Analysis of DEPs with Gene Ontology (GO) and Kyoto Encyclopedia of Genes and Genomes (KEGG)

**Figure 2:**
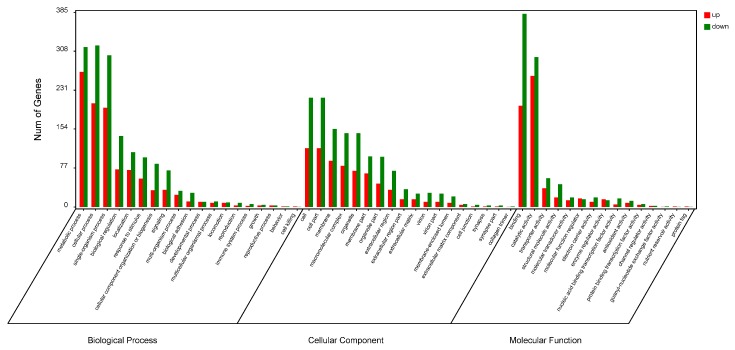
Enriched gene ontology (GO) analysis of differentially expressed proteins after metamorphosis. The most enriched GO terms (based on gene number) in “Cellular component,” “Molecular function,” and “Biological process” are shown.

Under GO analysis, significant enrichment (*p* < 0.05) was found for 77, 27, and 63 categories in the biological process (BP), cellular component (CC), and molecular function (MF) domains, respectively (Table S3). The most enriched GO terms were metabolic, cellular, and single-organism processes in BPs; cell and cell part in CCs; as well as binding and catalytic activity secondary items in MFs (Figure 2).

Of the 38 significantly enriched pathways under KEGG analysis (*p* < 0.05; Table S4), seven were reliably enriched after adjustment (*q* < 0.05; Table 3). The high representation of phototransduction, pentose and glucuronate, olfactory transduction, and salivary secretion pathways suggest changes to ingestion and digestion characteristics during metamorphosis. Additionally, enrichment in glycerolipid metabolism and galactose metabolism pathways illustrate differing energy strategies between competent larvae and post-larvae.

### 2.3. Association Analysis of Transcriptome and Proteome Data

**Figure 3:**
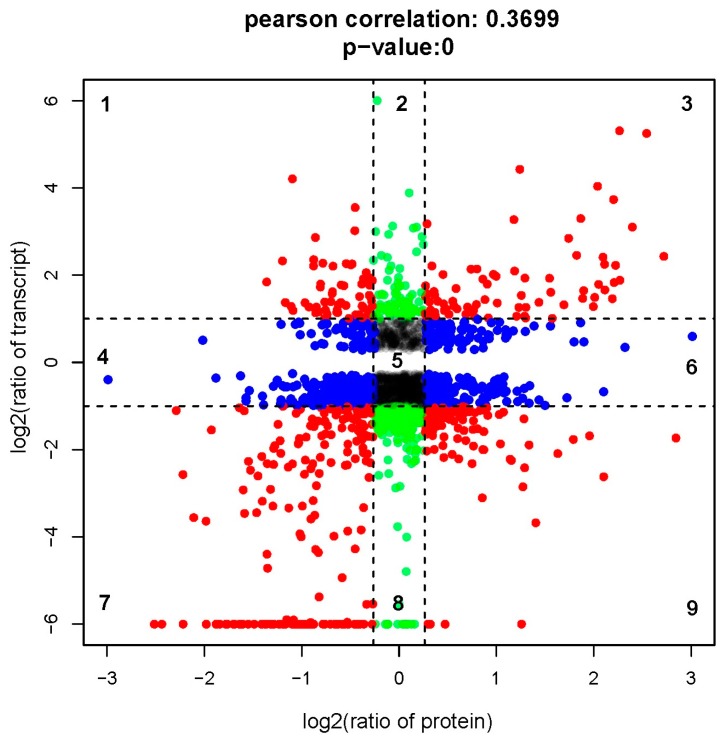
Comparison of expression ratios from transcriptomic (*y*-axis) and proteomic (*x*-axis) profiling. Log_2_ expression ratios were calculated from competent larvae *versus* post-larvae. Significant changes in expression are color-coded: **blue**, proteins only; **green**, transcripts only; **red**, both.

We performed a direct comparison of transcriptome and proteome abundance during metamorphosis. Concordance tests revealed a significant relationship between mRNA and protein ratios (Pearson’s correlation, *r* = 0.3699; Figure 3). We observed 458 concordant dots, representing a correspondence of protein abundance with transcript accumulation (red dots in Figure 3). We also found 282 green dots and 592 blue dots, respectively, indicating differential expression only on the transcript or the protein levels. Detailed quantitation and annotation on the points in Figure 3 are provided in Table S5.

## 3. Discussion

In this study, we performed a proteomic analysis to identify DEPs before and after *R. venosa* metamorphosis. Based on the reference transcriptome, we identified 470 upregulated proteins and 668 downregulated proteins. These DEPs were generally associated with cytoskeleton and cell adhesion, ingestion and digestion, immunity and stress response, transcription and translation, specific tissue development, and signal transduction. Additionally, their differential expression patterns reflect life-stage transitions in *R. venosa* (Table 2). We discuss the implications of our results in the following sections.

### 3.1. Cytoskeleton and Cell Adhesion

The intracellular cytoskeleton, transmembrane cell-adhesion components, and extracellular matrices (ECMs) comprise a complex “skeleton” network, which is critical for cell motility processes, including proliferation, differentiation, migration, and apoptosis. In this study, active cell motility during metamorphosis is indicated by the abundance of proteins involved in cytoskeleton, cell adhesion, and ECMs.

Tubulins (tubulin α-1 chain, tubulin α-2 chain, tubulin β-2 chain, and tubulin β-4B chain) were highly expressed in larvae but declined in post-larvae. As components of microtubules, alpha and beta tubulins function in essential cellular processes, including cell division, proliferation, and migration [19]. Any temporal variation in tubulin expression is likely related to various physiological functions and post-translational modifications [20,21]. Thus, the expression patterns that we observed are consistent with the suggestion that protein degradation and apoptosis during metamorphosis mediate the loss of larval organs, as well as the morphogenesis of juvenile characteristics [22,23]. Furthermore, our results conformed with studies in marine invertebrates (e.g., the spionid polychaete *Pseudopolydora vexillosa* [24] and polychaete *Hydroides elegans* [13]) that demonstrated a decline of tubulin isoforms during metamorphosis.

Proteins associated with ECMs were also differentially expressed. Specifically, we observed downregulation in collagen α-1 (XV, XXI, and XXII chain), collagen α-6 (VI chain), and matrix metalloproteinase-19. The ECM is the cell base and participates in tissue remodeling, as well as cell migration and differentiation; convincing evidence exists to show that ECMs are remodeled during metamorphic transition [25,26], and, in fact, the process is considered essential in the metamorphosis of amphibians [26,27], insects [28], and mollusks [29]. Thus, the observed expression patterns suggest that ECM remodeling—specifically involving the identified proteins—functions in *R. venosa* metamorphosis. Although this hypothesis requires further validation for our study species, we note that collagenase (a matrix-metalloprotease) was first discovered in the tail of a tadpole undergoing metamorphosis [30]. Additionally, matrix metalloprotease was highly expressed during the metamorphosis of the lepidopteran *Galleria mellonella*, causing collagen degradation [31].

### 3.2. Ingestion and Digestion

Morphological and functional changes in the digestive system clearly play a vital role in the metamorphic transition of *R. venosa* from a diet of microalgae to one of bivalve mollusks [7]. It follows that proteins associated with food intake and digestion will be differentially expressed between the larval and post-larval stages. Indeed, we found that post-metamorphosis, carnivorous digestive enzymes clearly increased, whereas phytophagous digestive enzymes were downregulated. Our study provides novel molecular data on the dietary shift that occurs with metamorphic transition.

In larval *R. venosa*, we detected several enzymes involved in the breakdown of cellulose and hemi-cellulose, both plant cell-wall components. Specifically, we observed two important cellulase components, endoglucanase and exoglucanase, as well as endo-1,4-β-xylanase, important in the hydrolysis of hemicellulose. Next, we also observed the presence of β-galactosidase, a key enzyme in the hydrolysis of lactose into galactose and glucose. Together, these data indicated that larval whelks were able to completely digest and absorb microalgae. High levels of cellulases have been reported in the pre-competent and competent larvae of the spotted babylon snail *Babylonia areolata,* which also has a pre-metamorphosis diet of microalgae [32], suggesting that the two species may have similar digestive mechanisms.

In *R. venosa* post-larvae, we observed higher levels of proteolytic enzymes, illustrating the capacity to exploit varied protein diets post-metamorphosis. For example, serine proteases (trypsin and chymotrypsin), as well as zinc carboxypeptidase, are major proteolytic enzymes in the gastropod digestive glands and were all highly expressed. Additionally, we observed an upregulation of pancreatic triacylglycerol lipase in post-larvae. Through hydrolysis, lipases prepare fatty acids for absorption through membranes [33]. Our results are corroborated by previous findings of high lipase expression in *B. areolata* juveniles [32]. Taken together, we suggest that cellulase downregulation and protease/lipase upregulation are primarily responsible for the transition from herbivores to carnivores in *R. venosa* with biphasic life history.

Unexpectedly, we found high expression of conotoxin and cysteine-rich venom protein in the post-larvae. The former is a neurotoxic peptide that was first isolated from the venom of the predatory marine cone snail (*Conus* spp.) [34]. The latter has also been found in a particular species of cone snail, *Conus textile*, where it exhibits protease activity and functions in pro-conotoxin processing of *C. textile* venom [35]. Our results suggest that *R. venosa* may possess predation mechanisms homologous to *Conus*. As little information is available regarding the composition and toxicity of *R. venosa* venom, the presence of conotoxin observed here warrants further research.

In summary, the diverse suite of proteins associated with ingestion and digestion illustrates the capacity of *R. venosa* to exploit different diets that suit the shifting nutritional requirements in a biphasic life cycle.

### 3.3. Stress Response and Immunity

Proteins involved in stress response and immunity tend to be upregulated during metamorphosis [36]. In the present study, we found that anti-oxidant enzymes, such as thioredoxin-T and peroxiredoxin-2, were highly expressed in the competent larval stage. Similarly, significant upregulation of peroxiredoxin has been documented in *Crassostrea gigas* post-metamorphosis [29]. These patterns suggest that competent larvae may experience considerable oxidative stress from reactive oxygen species (ROS) [29]. Indeed, amphibian studies have shown that when endogenous thyroid hormone induces metamorphosis, it also enhances mitochondrial respiration, which leads to higher ROS content [37,38]. Similar mechanisms may be at work in *R. venosa*, and the observed anti-oxidant enzymes are likely essential for protection against ROS-induced cell damage and maintenance of cell redox homeostasis during the metamorphosis.

We also noticed that *R. venosa* hemocyanin (RvH) A-type and RvH G-type were significantly upregulated after metamorphosis. Hemocyanin was first identified in the snail *Helix pomatia*; the protein has two copper atoms that reversibly bind with oxygen and acts as an oxygen transport molecule similar to hemoglobin. Under cold environments with low oxygen pressure, hemocyanin is more efficient at oxygen transportation than its vertebrate counterpart [39]. However, hemocyanin also plays important roles in innate immunity, exhibiting antiviral, antimicrobial, and antitumor activities [40,41]. Further evidence supporting this role in immune function includes the identification of four novel proline-rich peptides from RvH that exhibit antimicrobial activities against Gram-positive *Klebsiella pneumonia* and Gram-negative *Staphylococcus aureus* [42]. Moreover, the structural subunits RvH-1 and RvH2 exert strong antiviral effects upon the Herpes simplex virus [43,44]. Thus, two complementary levels of explanation could account for abundant RvH expression in juvenile *R. venosa*: on the evolutionary level, it is an adaptation to hypoxia stress at the benthic life stage, and on the developmental level, it reflects immune-system maturation post-metamorphosis. In support of the latter concept, proteins such as α-2macroglobulin and myeloperoxidase were also elevated in post-larvae. α-2-macroglobulins are selective protease inhibitors and major components of the eukaryotic innate immune system [45], while myeloperoxidase is highly expressed in neutrophil granulocytes, where it produces antimicrobial hypohalous acids [46].

### 3.4. Specific Tissue Development

Tissue-specific or tissue-preferential DEPs likely reflect physiological changes in those tissues [29]. For example, fluctuations in tropomyosin and myosin abundance are closely associated with muscle development during the metamorphosis of red abalone *Haliotis rufescens* [47,48]. Here, we demonstrated that larvae and post-larvae exhibit differential expression of neuron- and muscle-specific proteins, including myosin heavy chain, myosin light chain, and neuroglian proteins. All of these proteins are closely involved with transitions in nervous and muscular systems during molluscan metamorphosis [36,48].

As described earlier (see Section 3.1), tubulins were downregulated after metamorphosis. These proteins are cilia-specific, along with tektin, dynein heavy chain, and dynein beta chain, all of which experienced downregulation. The decline of proteins that comprise core cilial structure and function in cilia movement accords with post-metamorphic degradation of the velum, a conspicuous, ciliated organ in larvae used for swimming and filter-feeding.

## 4. Materials and Methods

### 4.1. Larvae Culture and Sample Collection

Egg capsules of *Rapana venosa* were obtained from rocks in Laizhou Bay (37°11′4.78″ N, 119°41′3.75″ E), Laizhou, China. Larvae were cultivated at Blue Ocean Co. Limited (Laizhou, China) following previously published methods [7]: pelagic larvae were cultured in 2.5 m × 2.5 m × 1.5 m tanks with a density range of 0.3–0.05 ind/mL, depending on developmental stage. Diets were a mixture of microalgae *Platymonas subcordiformis*, *Isochrysis galbana*, and *Chlorella vulgaris*; larvae were fed 13.0 × 10^4^ cell/mL daily. Seawater used for culturing was filtered with sand and radiosterilized with UV light. Water temperature was maintained below 25 ± 1 °C. Larvae samples from four spiral-whorl stages (competent larva) and post-larval stages were collected and examined under a microscope to guarantee developmental synchronies. Samples were immediately washed with dH_2_O, snap frozen in liquid nitrogen, stored at −80 °C till use.

### 4.2. Protein Extraction, Digestion, and iTRAQ Labelling

Three biological replicates (each containing approximately 500 mg larvae) were prepared for the iTRAQ analysis. Total proteins were extracted using the cold acetone method. Samples were ground to powder in liquid nitrogen before the addition of 2 mM EDTA and 1 mM PMSF, then dissolved in lysis buffer. After 5 min, DTT (10 mM) was added to the samples, which were centrifuged at 4 °C and 25,000× *g* for 20 min. All subsequent centrifugation steps described in this section occurred at 4 °C and 25,000× *g*. The precipitate was then discarded and the supernatant was mixed with 10 mM DTT in 5× volume of cold acetone, followed by incubation at −20 °C for 12 h. After a second round of centrifugation for 20 min, the supernatant was discarded. Pellets were washed in 1.5 mL cold acetone (containing 10 mM DTT), then centrifuged a third time for 15 min, to discard the supernatant. This final step was repeated three times. The precipitate was then air-dried and resuspended in 1 mL extraction buffer (10 mM DTT, 4% (*w*/*v*) CHAPS, 30 mM HEPES, 8 M urea, 1 mM PMSF and 2 mM EDTA), sonicated for 10 min, and centrifuged for 15 min. The resulting supernatant was transferred to a new tube, mixed with 10 mM DDT, and incubated at 56 °C for 1 h. The solution was incubated in a dark room for another hour after the addition of iodacetamide (55 mM), then precipitated in cold acetone at −20 °C overnight. Finally, the precipitate was centrifuged for 15 min, air-dried, and dissolved in 1 mL extraction buffer under ultrasound. Protein quality and concentrations were examined with SDS-PAGE and the 2-D Quant Kit (General Electric Company, Fairfield, CT, USA), respectively.

Protein digestion was performed with Trypsin Gold (Promega, Madison, WI, USA) for 16 h at 37 °C, and peptides were dried in a centrifugal vacuum concentrator. Competent-larvae samples were labeled with iTRAQ tags 113, 114, and 115, whereas post-larvae samples were labeled with tags 118, 119, and 121, following manufacturer protocol in the iTRAQ 8-plex labelling kit (Applied Biosystems, Foster City, CA, USA).

### 4.3. Strong Cation Exchange (SCX) Fractionation and Liquid Chromatography–Tandem Mass Spectrometry (LC-MS/MS) Analysis

Labeled samples were pooled and subjected to the SCX fractionation column connected with an HPLC system (LC-20ab, Shimadzu, Kyoto, Japan). Peptides were eluted using buffer-1 (25 mM NaH_2_PO_4_ in 25% ACN, pH 2.7) and a gradient of buffer-2 (25 mM NaH_2_PO_4_, 1 M KCl in 25% ACN, pH 2.7). The fractionating procedure was as follows: 100% buffer A for 10 min, 5%–35% buffer B for 20 min, 35%–80% buffer-2 for 1 min. Flow rate was kept at 1 mL/min. Fractions were desalted using a Strata X 33-μm Polymeric Reversed Phase column (Phenomenex, Torrance, CA, USA) and vacuum-dried.

Peptide fractions were analyzed using Nano HPLC (LC-20AD Shimadzu, Kyoto, Japan) and a 10-cm eluting C18 column (Shimadzu, Kyoto, Japan). A Triple TOF 5600 instrument (AB SCIEX, Concord, ON, Canada), fitted with Nanospray III (AB SCIEX) and a pulled quartz-tip emitter (New Objectives, Woburn, MA, USA), was used for mass spectrometry [49]. This procedure was carried out by Guangzhou Gene denovo Biotechnology Co., Ltd. (Guangzhou, China).

### 4.4. Protein Identification and Quantification

Raw data from LC-MS/MS were transformed into MGF files with Proteome Discovery 1.2 (Thermo, Pittsburgh, PA, USA). In the Mascot search engine (version 2.3.02, Matrix, Science, London, UK), proteins were identified using the *R. venosa* reference transcriptome [9]. Mascot search results were then normalized and quantified. Proteins with fold changes significantly (*p* < 0.05) >1.2 or <0.83 were considered differentially expressed [49].

### 4.5. Enrichment of GO and KEGG Pathways

We searched against the GO and KEGG databases to classify and identify differentially expressed proteins [50,51]. Significant pathway enrichment was examined with the hypergeometric test, and significance was set at *p* < 0.05.

### 4.6. Correlation Analysis of Transcriptomic and Proteomic Data

Previously, we had constructed an RNA-seq library of competent larvae and post-larvae (raw data available in NCBI GEO, accession number GSE70548). To investigate the concordance between transcript and protein levels, we calculated the Pearson’s correlation for these data and created scatterplots with the expression ratios of competent larvae *versus* post-larvae.

## 5. Conclusions

Using iTRAQ, we constructed a comprehensive and quantitative proteomic profile of *R. venosa* larvae and post-larvae. To our knowledge, this work is the first proteomic study focused on gastropod metamorphosis. We identified over a thousand differentially expressed proteins that reflected physiological processes occurring in metamorphosis, including changes to cytoskeleton and cell adhesion, ingestion and digestion, stress response and immunity, as well as tissue development. Our data contributed to a better understanding of the regulatory mechanisms underlying *R. venosa* development through identifying major participating proteins. Therefore, this study should provide a sound basis for future studies aiming to investigate specific metamorphosis-related proteins in greater depth.

## Figures and Tables

**Table 1:** Overview of proteomics sequencing results.

Item	Value
Total Spectra	224,473
Spectra	53,723
Unique Spectra	46,485
Peptide	21,626
Unique Peptide	20,175
Protein	5312
Upregulated protein	470
Downregulated protein	668

**Table 2:** Selected differentially expressed proteins (DEPs) between competent larvae and post-larvae. “FC” represents Log_2_ (competent larvae /post larvae).

Accession	FC	*p*-Value	Annotation	Organism Species	Description
Cytoskeleton and Cell Adhesion					
c111395_g1	0.49	8.22 × 10^−3^	Paramyosin	*Mytilus galloprovincialis*	cytoskeleton component
c119060_g1	1.01	6.45 × 10^−5^	Paramyosin	*Mytilus galloprovincialis*	cytoskeleton component
c67246_g1	0.80	1.29 × 10^−3^	Paramyosin	*Mytilus galloprovincialis*	cytoskeleton component
c128871_g1	0.30	3.16 × 10^−2^	Tropomyosin-2	*Biomphalaria glabrata*	cytoskeleton component
c128871_g1	0.30	3.16 × 10^−2^	Tropomyosin-2	*Biomphalaria glabrata*	cytoskeleton component
c64757_g1	1.42	1.03 × 10^−4^	Tubulin α chain	*Plasmodium falciparum*	cytoskeleton component
c144449_g1	−0.63	1.02 × 10^−2^	Tubulin α-1 chain	*Paracentrotus lividus*	cytoskeleton component
c19674_g1	−0.46	1.08 × 10^−2^	Tubulin α-2 chain	*Gossypium hirsutum*	cytoskeleton component
c65878_g1	0.60	5.21 × 10^−3^	Tubulin α-8 chain (Fragment)	*Gallus gallus*	cytoskeleton component
c129550_g1	−0.52	2.78 × 10^−2^	Tubulin β chain (Fragment)	*Haliotis discus*	cytoskeleton component
c52663_g1	−0.59	3.64 × 10^−3^	Tubulin β-2 chain	*Drosophila melanogaster*	cytoskeleton component
c91498_g1	−0.52	1.39 × 10^−3^	Tubulin β-4B chain	*Mesocricetus auratus*	cytoskeleton component
c154903_g1	−0.46	2.80 × 10^−4^	Collagen α-1(XV) chain	*Homo sapiens*	extracellular matrix
c136294_g1	−1.19	5.14 × 10^−3^	Collagen α-1(XXI) chain	*Xenopus laevis*	extracellular matrix
c156326_g1	−1.23	1.42 × 10^−4^	Collagen α-1(XXII) chain	*Homo sapiens*	extracellular matrix
c155801_g1	−0.56	1.18 × 10^−3^	Collagen α-4(VI) chain	*Crassostrea gigas*	extracellular matrix
c156014_g6	−0.85	1.72 × 10^−2^	Collagen α-5(VI) chain	*Crassostrea gigas*	extracellular matrix
c154603_g1	−0.91	2.20 × 10^−4^	Collagen α-6(VI) chain	*Homo sapiens*	extracellular matrix
c156014_g2	−1.06	9.95 × 10^−6^	Collagen α-6(VI) chain	*Homo sapiens*	extracellular matrix
c169434_g1	0.81	1.38 × 10^−2^	Extracellular matrix protein 3	*Lytechinus variegatus*	extracellular matrix
c215931_g1	0.87	2.65 × 10^−2^	FRAS1-related extracellular matrix protein 2	*Homo sapiens*	extracellular matrix
c157006_g5	−0.61	2.06 × 10^−2^	Laminin subunit alpha-2	*Mus musculus*	extracellular matrix
c155563_g1	−0.87	2.04 × 10^−4^	Laminin-like protein epi-1	*Crassostrea gigas*	extracellular matrix
c154307_g2	−0.79	4.65 × 10^−2^	Matrix metalloproteinase-19	*Homo sapiens*	extracellular matrix
c147589_g2	−0.89	1.45 × 10^−2^	Cadherin-89D	*Drosophila melanogaster*	involved in adhesion
c149462_g1	0.42	9.10 × 10^−3^	Kinectin	*Mus musculus*	involved in adhesion
c104353_g1	−0.55	2.02 × 10^−2^	Lactadherin	*Rattus norvegicus*	involved in adhesion
c156870_g1	0.64	8.39 × 10^−4^	Macrophage mannose receptor 1	*Homo sapiens*	involved in adhesion
c156842_g1	−0.36	1.64 × 10^−3^	Neural cell adhesion molecule 1	*Bos taurus*	involved in adhesion
c151606_g1	−0.40	6.31 × 10^−3^	Neural cell adhesion molecule 1	*Rattus norvegicus*	involved in adhesion
c136200_g1	−0.31	2.49 × 10^−2^	Neuroglian	*Drosophila melanogaster*	involved in adhesion
c154303_g4	0.68	2.35 × 10^−2^	Non-neuronal cytoplasmic intermediate filament protein	*Helix aspersa*	involved in adhesion
c135777_g1	−1.18	5.99 × 10^−5^	Periostin	*Mus musculus*	involved in adhesion
c157397_g1	−1.38	1.25 × 10^−5^	Protocadherin Fat 4	*Homo sapiens*	involved in adhesion
c142570_g1	−1.11	2.18 × 10^−4^	Protocadherin-like wing polarity protein stan	*Drosophila melanogaster*	involved in adhesion
Ingestion and Digestion					
c128401_g2	−0.78	2.31 × 10^−2^	Beta-galactosidase-1-like protein 2	*Homo sapiens*	involved in carbohydrates hydrolysis
c135558_g1	−1.78	1.09 × 10^−3^	Endo-1,4-β-xylanase Z	*Clostridium thermocellum*	involved in carbohydrates hydrolysis
c96519_g1	−1.58	5.17 × 10^−3^	Endoglucanase	*Mytilus edulis*	involved in carbohydrates hydrolysis
c137870_g1	−1.17	1.63 × 10^−3^	Endoglucanase E-4	*Thermobifida fusca*	involved in carbohydrates hydrolysis
c154739_g1	−0.98	7.49 × 10^−4^	Endoglucanase E-4	*Thermobifida fusca*	involved in carbohydrates hydrolysis
c150903_g1	−1.78	1.09 × 10^−3^	Exoglucanase XynX	*Clostridium thermocellum*	involved in carbohydrates hydrolysis
c145604_g1	1.18	6.57 × 10^−5^	Inactive pancreatic lipase-related protein 1	*Rattus norvegicus*	involved in fat hydrolysis
c71768_g2	2.20	5.20 × 10^−7^	Pancreatic triacylglycerol lipase	*Myocastor coypus*	involved in fat hydrolysis
c141966_g1	1.21	2.76 × 10^−3^	Chymotrypsin-like elastase family member 3B	*Mus musculus*	involved in proteins hydrolysis
c140662_g1	0.74	3.47 × 10^−3^	Chymotrypsin-like serine proteinase	*Haliotis rufescens*	involved in proteins hydrolysis
c141241_g2	0.33	2.94 × 10^−2^	Glutamate carboxypeptidase 2	*Rattus norvegicus*	involved in proteins hydrolysis
c150838_g1	1.44	4.46 × 10^−5^	Prolyl endopeptidase	*Mus musculus*	involved in proteins hydrolysis
c153823_g1	0.43	3.91 × 10^−3^	Trypsin	*Sus scrofa*	involved in proteins hydrolysis
c149315_g1	1.96	4.51 × 10^−4^	Zinc carboxypeptidase A 1	*Anopheles gambiae*	involved in proteins hydrolysis
c150282_g1	2.22	2.11 × 10^−4^	Zinc metalloproteinase nas-13	*Caenorhabditis elegans*	involved in proteins hydrolysis
c146629_g1	1.74	3.63 × 10^−4^	Zinc metalloproteinase nas-14	*Caenorhabditis elegans*	involved in proteins hydrolysis
c149138_g1	−0.45	3.15 × 10^−3^	Zinc metalloproteinase nas-30	*Caenorhabditis elegans*	involved in proteins hydrolysis
c128907_g1	1.87	1.72 × 10^−3^	Zinc metalloproteinase nas-38	*Caenorhabditis elegans*	involved in proteins hydrolysis
c153700_g1	1.79	1.27 × 10^−4^	Zinc metalloproteinase nas-6	*Caenorhabditis elegans*	involved in proteins hydrolysis
c156669_g2	2.30	3.77 × 10^−5^	Zinc metalloproteinase nas-8	*Caenorhabditis elegans*	involved in proteins hydrolysis
c131553_g1	0.83	4.63 × 10^−3^	Conotoxin Cl14.12	*Conus californicus*	involved in secretory venom for predation
c147316_g1	1.33	9.47 × 10^−4^	Cysteine-rich venom protein	*Conus textile*	involved in secretory venom for predation
c143655_g1	2.27	2.96 × 10^−4^	Cysteine-rich venom protein Mr30	*Conus marmoreus*	involved in secretory venom for predation
Stress Response and Immunity					
c122242_g1	1.59	1.84 × 10^−4^	Myeloperoxidase	*Mus musculus*	anti-oxidant protein
c88819_g1	1.68	1.13 × 10^−3^	Peroxidase-like protein 3 (Fragment)	*Lottia gigantea*	anti-oxidant protein
c156674_g2	0.49	1.21 × 10^−2^	Peroxidasin homolog	*Mus musculus*	anti-oxidant protein
c140657_g1	−0.38	1.54 × 10^−2^	Peroxiredoxin-2	*Rattus norvegicus*	anti-oxidant protein
c142245_g1	−0.37	1.23 × 10^−2^	Peroxiredoxin-6	*Gallus gallus*	anti-oxidant protein
c156482_g1	0.30	1.01 × 10^−2^	Probable deferrochelatase/peroxidase YfeX	*Escherichia coli*	anti-oxidant protein
c130129_g1	−0.69	3.65 × 10^−3^	Thioredoxin-T	*Drosophila melanogaster*	anti-oxidant protein
c152296_g4	−0.85	1.93 × 10^−3^	Angiotensin-converting enzyme (Fragment)	*Gallus gallus*	immune-related protein
c154571_g1	−2.22	1.81 × 10^−3^	Uncharacterized protein C1orf194 homolog	*Danio rerio*	immune-related protein
c120194_g1	2.15	5.35 × 10^−5^	Hemocyanin A-type, units Ode to Odg (Fragment)	*Enteroctopus dofleini*	oxygen supply, immune-related protein
c147531_g1	2.31	2.43 × 10^−5^	Hemocyanin A-type, units Ode to Odg (Fragment)	*Enteroctopus dofleini*	oxygen supply, immune-related protein
c153812_g1	2.41	2.00 × 10^−4^	Hemocyanin G-type, units Oda to Odg	*Enteroctopus dofleini*	oxygen supply, immune-related protein
c146636_g1	2.42	1.95 × 10^−4^	Hemocyanin G-type, units Oda to Odg	*Enteroctopus dofleini*	oxygen supply, immune-related protein
c156294_g1	2.43	7.60 × 10^−5^	Hemocyanin G-type, units Oda to Odg	*Enteroctopus dofleini*	oxygen supply, immune-related protein
c153794_g2	1.02	1.50 × 10^−3^	Alpha-2-macroglobulin	*Pongo abelii*	proteolysis, immune-related protein
c155750_g1	0.45	5.33 × 10^−5^	60 kDa heat shock protein, mitochondrial	*Cricetulus griseus*	response to stress
c155284_g2	0.42	7.02 × 10^−3^	Heat shock protein 75 kDa, mitochondrial	*Mus musculus*	response to stress
Particular Tissue Development					
c157271_g1	−0.73	2.29 × 10^−2^	Dynein heavy chain 10, axonemal	*Strongylocentrotus purpuratus*	cilia-specific protein
c156807_g2	−0.76	1.09 × 10^−2^	Dynein heavy chain 12, axonemal	*Xenopus laevis*	cilia-specific protein
c123013_g1	−0.56	3.64 × 10^−2^	Dynein heavy chain 5, axonemal	*Bos taurus*	cilia-specific protein
c155384_g3	−0.64	2.82 × 10^−3^	Dynein heavy chain 6, axonemal	*Rattus norvegicus*	cilia-specific protein
c154803_g2	−0.76	4.90 × 10^−3^	Dynein heavy chain 7, axonemal	*Homo sapiens*	cilia-specific protein
c157287_g2	−0.79	2.31 × 10^−4^	Dynein heavy chain 8, axonema	*Mus musculus*	cilia-specific protein
c154991_g1	−1.02	5.78 × 10^−4^	Dynein intermediate chain 2, ciliary	*Heliocidaris crassispina*	cilia-specific protein
c122667_g1	−0.87	1.98 × 10^−3^	Dynein light chain 1, axonemal	*Homo sapiens*	cilia-specific protein
c156053_g3	0.89	2.24 × 10^−3^	Myosin essential light chain, striated adductor muscle	*Homo sapiens*	cilia-specific protein
c85433_g2	0.89	8.64 × 10^−4^	Myosin heavy chain, striated muscle	*Homo sapiens*	cilia-specific protein
c151606_g1	−0.40	6.31 × 10^−3^	Neural cell adhesion molecule 1	*Homo sapiens*	cilia-specific protein
c136200_g1	−0.31	2.49 × 10^−2^	Neuroglian	*Homo sapiens*	cilia-specific protein
c150230_g2	−1.42	7.67 × 10^−6^	Tektin-1	*Homo sapiens*	cilia-specific protein
c153806_g1	−1.58	2.39 × 10^−4^	Tektin-2	*Homo sapiens*	cilia-specific protein
c155866_g1	−1.30	2.54 × 10^−4^	Tektin-3	*Rattus norvegicus*	cilia-specific protein
c28062_g1	−0.89	1.80 × 10^−4^	Tektin-4	*Tripneustes gratilla*	cilia-specific protein
c153806_g3	−1.19	1.10 × 10^−4^	Tektin-B1	*Heliocidaris crassispina*	cilia-specific protein
c131813_g1	−1.08	1.05 × 10^−2^	Dynein beta chain, ciliary	*Argopecten irradians*	muscle-specific protein
c95355_g1	−0.93	4.43 × 10^−3^	Dynein beta chain, ciliary	*Argopecten irradians*	muscle-specific protein
c157057_g1	−0.68	1.06 × 10^−2^	Dynein heavy chain 1, axonemal	*Drosophila melanogaster*	neuron-specific protein
c155993_g1	−0.64	4.95 × 10^−3^	Dynein heavy chain 10, axonemal	*Rattus norvegicus*	neuron-specific protein

**Table 3:** Seven enriched pathways identified with KEGG analysis of differentially expressed proteins.

#	Pathway	Differential Proteins with Pathway Annotation (347)	All Proteins with Pathway Annotation (2056)	*p*-Value	*q*-Value	Pathway ID
1	Phototransduction	7 (2.02%)	11 (0.54%)	0.000655	0.039412	ko04744
2	Caprolactam degradation	7 (2.02%)	11 (0.54%)	0.000655	0.039412	ko00930
3	Pentose and glucuronate Interconversions	12 (3.46%)	27 (1.31%)	0.000685	0.039412	ko00040
4	Olfactory transduction	9 (2.59%)	18 (0.88%)	0.001175	0.039412	ko04740
5	Glycerolipid metabolism	11 (3.17%)	25 (1.22%)	0.001275	0.039412	ko00561
6	Galactose metabolism	11 (3.17%)	25 (1.22%)	0.001275	0.039412	ko00052
7	Salivary secretion	17 (4.9%)	48 (2.33%)	0.001326	0.039412	ko04970

## Supplementary Materials

Supplementary materials can be found at http://www.mdpi.com/1422-0067/17/6/924/s1.

## Abbreviations

2DE	Two-Dimensional Electrophoresis
ACN	Acetonitrile
CHAPS	3-[(3-cholamidopropyl)dimethylammonio]propanesulfonate
DEPs	Differentially Expressed Proteins
DTT	DL-Dithiothreitol
ECM	Extracellular Matrix
EDTA	Ethylene Diamine Tetraacetic Acid
GO	Gene Ontology
HEPES	4-(2-hydroxyethyl)-1-piperazineethanesulfonic Acid
HPLC	High Performance Liquid Chromatography
iTRAQ	isobaric Tags for Relative and Absolute Quantitation
KEGG	Kyoto Encyclopedia of Genes and Genomes
LC–MS/MS	Fractionation and Liquid Chromatography–Tandem Mass Spectrometry
PMSF	Phenylmethanesulfonyl Fluoride
SCX	Strong Cation Exchange
SDS-PAGE	Sodium Dodecyl Sulfate-Polyacrylamide Gel Electrophoresis
